# Endovascular thrombectomy in a patient with ischemic stroke and vertebrobasilar dolichoectasia: A case report

**DOI:** 10.1097/MD.0000000000045198

**Published:** 2025-10-24

**Authors:** Shuang Guo, Weimin Zhang, Xuandong Jiang, Wenchen Li

**Affiliations:** a Intensive Care Unit, Affiliated Dongyang Hospital of Wenzhou Medical University, Dongyang, Zhejiang Province, P.R. China; b Neurology, Affiliated Dongyang Hospital of Wenzhou Medical University, Dongyang, Zhejiang Province, P.R. China.

**Keywords:** endovascular thrombectomy, endovascular treatment, ischemic stroke, vertebrobasilar dolichoectasia

## Abstract

**Rationale:**

Vertebrobasilar dolichoectasia (VBD) is a rare vascular disorder characterized by the dilation, elongation, and tortuosity of the vertebrobasilar artery; a condition which tends to culminate in an ischemic stroke. In cases of VBD complicated by ischemic strokes, reperfusion therapies include chemical thrombolysis and mechanical endovascular thrombectomy. The efficacy of endovascular therapy compared to that of pharmacological treatment in VBD-related ischemic stroke remains unclear. In this study, we present the case of a patient with VBD complicated by ischemic stroke who underwent thrombectomy, shedding light on the complexities and considerations involved in managing this condition.

**Patient concerns:**

This report presents a case of a 59-year-old male patient admitted with speech difficulties and altered perceptual capabilities. It is worth noting that this patient had a history of hypertension.

**Diagnoses:**

Cranial and cervical computed tomography angiography (CTA) revealed aneurysmal dilation of the basilar artery with a filling defect, indicative of formation of a mural thrombus.

**Interventions:**

The patient underwent endovascular thrombectomy.

**Outcomes:**

The patient succumbed to brainstem and bilateral cerebellar hemisphere infarctions, as well as obstructive hydrocephalus.

**Lessons:**

VBD is a rare condition, with ischemic stroke serving as its most common clinical presentation and cause of death. Although the superiority of endovascular therapy over thrombolysis for acute ischemic stroke caused by VBD is not yet established, endovascular interventions are recommended for patients presenting with moderate to severe symptoms. Overall, larger-scale clinical trials are essential to evaluate the safety and effectiveness of endovascular treatment for basilar artery occlusion.

## 1. Introduction

Vertebrobasilar dolichoectasia (VBD) is a rare vascular condition characterized by the dilation, elongation, and tortuosity of the vertebrobasilar artery. The underlying pathophysiological mechanisms of VBD remain incompletely understood. However, its development is believed to involve a complex interplay of factors, including congenital abnormalities, infections, immune dysregulation, and degenerative processes. These factors are frequently associated with conditions such as atherosclerosis, hypertension, diabetes, hyperlipidemia, heart disease, stroke, transient ischemic attacks, carotid artery disease, and peripheral vascular disease.^[1,2]^

The clinical manifestations of VBD are varied, with ischemic stroke being the most common presentation and the leading cause of mortality.^[3]^ Emerging evidence highlight the significant role of VBD in posterior circulation ischemic strokes.^[4]^ Additional complications include hemorrhagic stroke, transient ischemic attacks, hydrocephalus, and symptoms resulting from brainstem and cranial nerve compression.^[5,6]^

Management of VBD complicated by ischemic stroke is challenging and carries a poor prognosis. Using antiplatelet or anticoagulant therapies poses a risk of cerebral hemorrhage.^[7]^ Endovascular intervention is another therapeutic option, though its efficacy and safety remain areas of active investigation.

In this report, we present the case of a patient with VBD complicated by ischemic stroke who underwent thrombectomy, shedding light on the complexities and considerations involved in managing this condition.

## 2. Case report

A 59-year-old male patient presented to the hospital with speech difficulties persisting for 4 hours, which progressed to altered consciousness over the subsequent 2 hours. His medical history included untreated hypertension with no history of diabetes, coronary heart disease, or hereditary disorders. The speech difficulties were characterized by expressive aphasia with preserved comprehension and were accompanied by motor dysfunction in the left limbs, manifesting as a limp and clumsiness in the left upper extremity.

**Figure 1.**
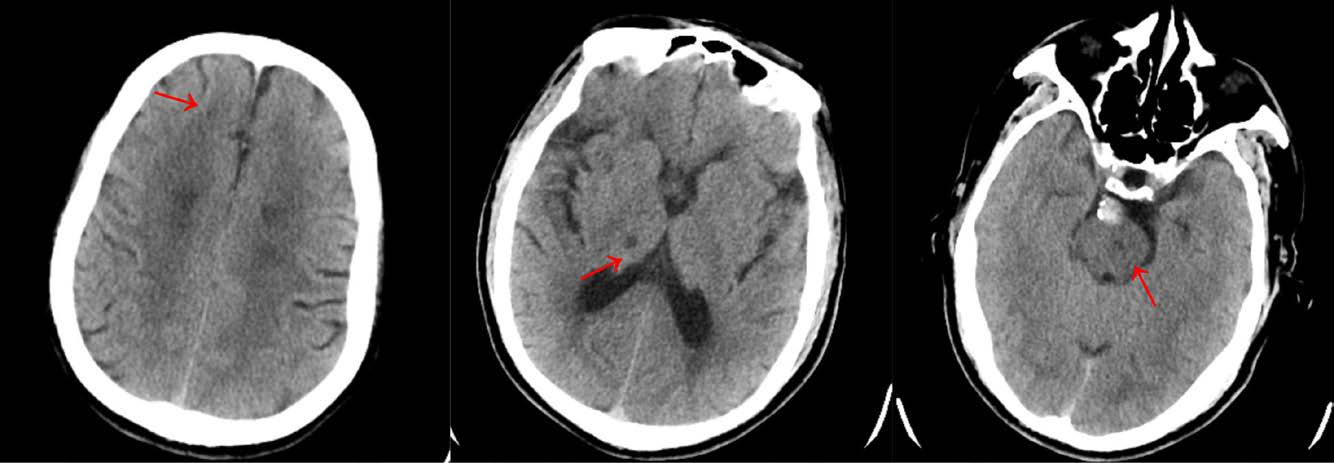
Cranial CT showing multiple lacunar infarctions in the bilateral frontal and parietal lobes, basal ganglia, thalamus, and brainstem. CT = computed tomography.

Cranial computed tomography (CT) revealed multiple lacunar infarctions in the bilateral frontal and parietal lobes, basal ganglia, thalamus, and brainstem (Fig. 1). Cranial and cervical CT angiography (CTA) revealed aneurysmal dilation of the basilar artery with a filling defect, suggestive of mural thrombus formation. The initial segment of the right vertebral artery exhibited thickening and calcification, causing moderate to severe luminal stenosis.

Two hours after symptom onset, the patient experienced a marked reduction in limb movement and altered consciousness. Although he could open his eyes, he did not respond to verbal stimuli. Physical examination revealed a blood pressure of 169/85 mm Hg and a National Institutes of Health Stroke Scale (NIHSS) score of 19. The modified Rankin Scale score was 0 (no symptoms) prior to stroke onset and 5 (severe disability, requiring constant care) upon admission. The patient appeared confused and uncooperative during verbal communication. Both pupils were equal and round at 3 mm in diameter; the left pupil responded briskly to light, while the right pupil’s response was sluggish. A leftward gaze was observed without spontaneous nystagmus. The bilateral nasolabial folds and forehead wrinkles were symmetrical, and no neck resistance was noted. Muscle strength assessments were limited owing to uncooperativeness, but flexion was observed in response to painful stimuli. Bilateral Babinski signs were positive.

The admission diagnoses included cerebral infarction, basilar artery thrombosis, primary hypertension (high risk), basilar artery malformation (VBD), and severe stenosis of the initial segment of the right vertebral artery.

**Figure 2.**
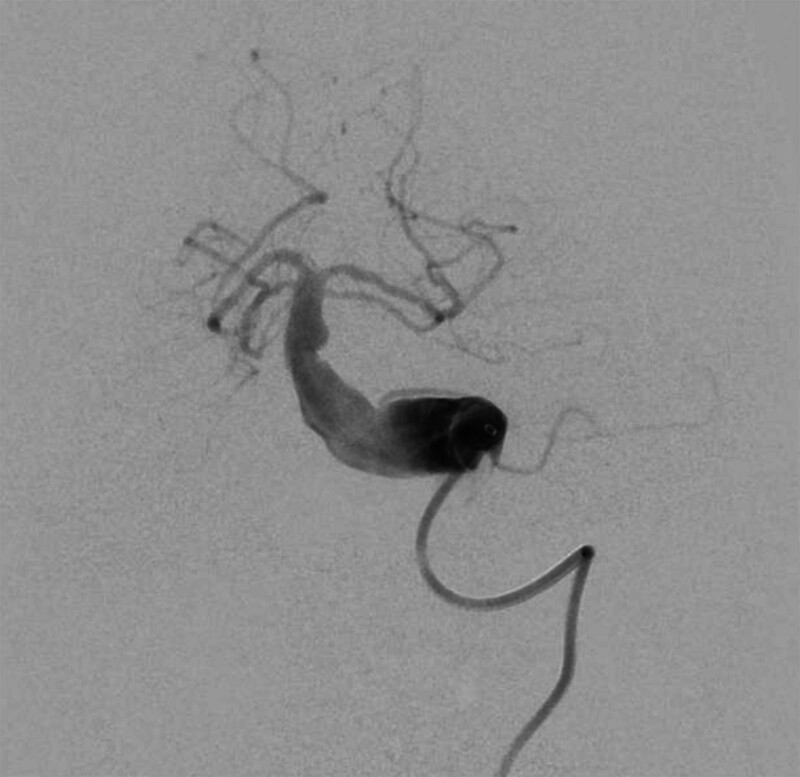
DSA images illustrating the aneurysmal dilation of the basilar artery and the V4 segments, with evidence of mural thrombus and localized blood stasis, highlighting the vascular abnormalities associated with VBD. DSA = digital subtraction angiography, VBD = vertebrobasilar dolichoectasia.

Endovascular therapy (EVT) was indicated due to the following: NIHSS = 19 reflecting catastrophic deficit, CTA/digital subtraction angiography (DSA) confirming basilar thrombus, and no CT evidence of completed brainstem infarction, consistent with guidelines for posterior circulation EVT within 24 hours. Endovascular intervention was initiated using a 6-F catheter advanced into the left vertebral artery. Angiography revealed elongation and dilation of the V4 segments of the left vertebral and basilar arteries, with localized blood stasis. A microcatheter was advanced to the distal basilar artery, and multiple aspirations using an intermediate catheter yielded a small thrombus. Post-intervention, distal basilar artery blood flow improved to thrombolysis in cerebral infarction grade 3. Bilateral common carotid artery angiography showed no significant large-vessel occlusion, whereas right vertebral artery angiography confirmed elongation and dilation of the V4 segment with turbulent blood flow but no significant occlusion (Fig. 2). The DSA procedure lasted 48 minutes.

The patient was admitted to the intensive care unit (ICU) for monitoring. Mild hypothermia and mechanical ventilation were initiated. Treatment included daily administration of enteric-coated aspirin (100 mg) via nasogastric tube, intravenous mannitol (100 mL) every 8 hours, edaravone-dexborneol concentrated solution (15 mL; edaravone 10 mg/dexborneol 2.5 mg) via intravenous drip twice daily, atorvastatin calcium (20 mg) via nasogastric tube nightly, and piperacillin/tazobactam (4.5 g) intravenously every 8 hours.

**Figure 3.**
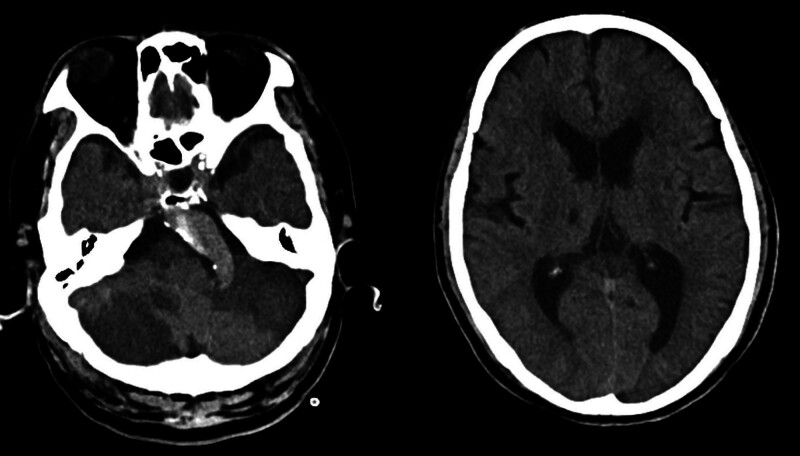
Cranial CT on day 2 after endovascular thrombectomy showing brainstem and bilateral cerebellar hemisphere infarctions with marked localized edema. CT = computed tomography.

**Figure 4.**
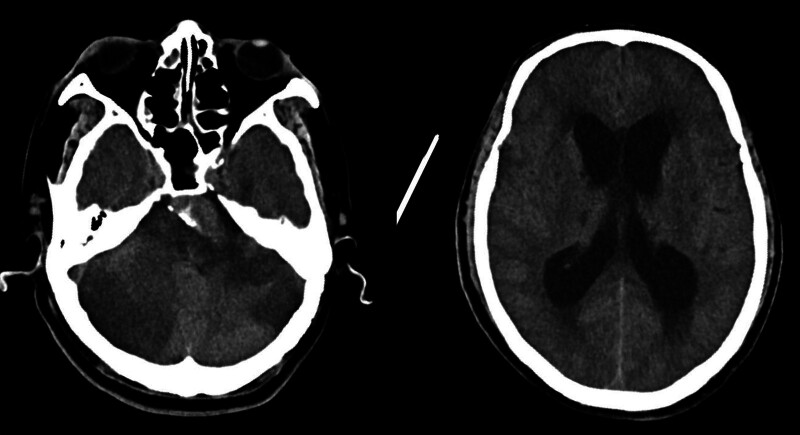
Cranial CT on day 4 revealing the worsening of infarctions in the brainstem and cerebellar hemispheres, with obstructive hydrocephalus. CT = computed tomography.

On day 2, the patient remained unconscious, with a Glasgow Coma Scale (GCS) score of E4VTM4. A repeat cranial CT scan revealed infarctions in the brainstem and bilateral cerebellar hemispheres with significant localized edema (Fig. 3). By day 4, the patient was in a deep coma with a GCS score of 3 and no cough reflex. Repeat cranial CT indicated progression of cerebral infarction with obstructive hydrocephalus (Fig. 4). Intravenous mannitol (100 mL every 8 hours) was administered to mitigate intracranial pressure. Given the patient’s GCS score of 3, absence of cough reflex, and lack of spontaneous breathing, surgical intervention was withheld.

On day 6, following the family’s decision to withdraw mechanical ventilation and discontinue treatment, the patient passed away.

## 3. Discussion

VBD is a rare vascular condition characterized by significant dilation, elongation, and tortuosity of the vertebrobasilar artery. Its development is believed to result from a combination of congenital factors, infections, immune system alterations, and degenerative conditions. Potential mechanisms include hypertension-induced atherosclerosis, congenital disorders such as autosomal recessive polycystic kidney disease, Pompe disease, and sickle cell anemia, as well as infections like syphilis.^[5]^ Although VBD could remain asymptomatic, symptomatic cases present with diverse clinical manifestations. The most common presentation is ischemic stroke, followed by brainstem and cranial nerve compression, hydrocephalus, and cerebral hemorrhage.^[3,5,6]^ In the present case, the patient’s irregularly managed hypertension contributed to atherosclerosis and unique hemodynamic changes associated with VBD, culminating in ischemic stroke. Infarction in the basilar artery territory resulted in secondary cerebral edema and obstructive hydrocephalus, ultimately leading to death.

The diagnosis of VBD relies heavily on imaging studies, supported by clinical findings. Current imaging modalities include CT, magnetic resonance imaging (MRI), and DSA. Smoker et al^[8]^ established diagnostic criteria based on CT and CTA, which remain widely used. According to these criteria, VBD is defined by a basilar artery bifurcation height score of ≥ 2, a positional laterality score of ≥ 2, and a diameter of ≥ 4.5 mm.^[8]^ Ubogu and Zaidat later proposed MRI-based criteria, defining dilation as a basilar artery diameter of > 4.5 mm, elongation as a basilar artery length ≥ 29.5 mm or lateral displacement of > 10 mm, and vertebral artery intracranial segment length of ≥ 23.5 mm or branch displacement of > 10 mm.^[9]^ CT and MRI also effectively assess other relevant features, including aneurysm formation, intraluminal thrombus or calcification, parenchymal changes, and the relationship between dilated arteries and cranial nerves. Although DSA remains the gold standard for diagnosing vascular diseases, specific diagnostic criteria for VBD using DSA are not yet established. In this case, DSA revealed localized blood stasis and vortices in the V4 segment of the left vertebral artery and the proximal basilar artery, indicating hemodynamic changes that promote thrombus formation and detachment. The elongation and displacement of the vertebrobasilar artery likely caused stretching of branch arteries, reducing blood perfusion.

Currently, no specific treatments are available to prevent arterial dilation in VBD beyond controlling blood pressure, blood sugar, and lipid levels.^[1]^ Treatment primarily focuses on managing symptoms and complications. For patients with VBD complicated by ischemic stroke, anticoagulants or antiplatelet agents are used to reduce the risk of recurrent strokes.^[10]^ However, these therapies may increase the risk of intracranial hemorrhage due to associated microbleeds.^[1]^ The risks and benefits of anticoagulant or antiplatelet therapy must be carefully evaluated, particularly during the acute phase of ischemic stroke.^[11]^ In the present case, the patient underwent arterial thrombectomy and received comprehensive ICU treatment but developed brainstem and bilateral cerebellar hemisphere infarctions with obstructive hydrocephalus, leading to a fatal outcome.

Reperfusion therapies, including chemical thrombolysis and endovascular mechanical thrombectomy, are the main treatments for acute basilar artery occlusion. For patients with progressive posterior circulation infarction caused by vertebrobasilar artery occlusion, EVT remains safe and effective even when the onset-to-puncture time exceeds 24 hours. Although no significant differences in functional outcomes or mortality have been observed between endovascular and pharmacological treatments, EVT has demonstrated significant advantages.^[12,13]^ Recent evidence demonstrates that, compared to standard medical treatment alone, EVT significantly reduces overall disability and mortality in patients with moderate to severe vertebrobasilar artery occlusion. However, this approach carries an increased risk of symptomatic intracranial hemorrhage. Furthermore, the benefit of endovascular thrombectomy remains uncertain in patients presenting with mild stroke severity and extensive infarcts on neuroimaging.^[14–16]^ EVT is recommended for patients with an NIHSS score of ≥ 6, a Posterior Circulation Alberta Stroke Program Early CT Score of ≥ 6, and an age between 18 and 89 years.^[17]^ Puncture-to-recanalization time, ASPECT score, DSA-ASITN collateral grading, and 24-hour NIHSS score may serve as important indicators for predicting outcomes in VBD patients undergoing EVT.^[18,19]^ Following reperfusion therapy for cerebral infarction, reperfusion injury may occur. The primary causes of death include malignant cerebral edema, pneumonia, and symptomatic intracranial hemorrhage.^[20]^ In the present case, the patient developed postprocedure edema in the brainstem and cerebellum, concurrently complicated by obstructive hydrocephalus, which ultimately contributed to the fatal outcome.

In conclusion, VBD is a rare condition with an unclear pathogenesis and a wide range of clinical presentations. Ischemic stroke is the most common manifestation and the leading cause of mortality in VBD. Although EVT is recommended for moderate to severe cases, its comparative benefits over thrombolytic therapy for ischemic stroke caused by VBD remain uncertain. Larger-scale trials are essential to confirm the efficacy and safety of EVT for basilar artery occlusion.

## Acknowledgments

We would like to thank Editage (www.editage.cn) for English language editing.

## Author contributions

**Conceptualization:** Weimin Zhang, Wenchen Li.

**Investigation:** Shuang Guo.

**Resources:** Shuang Guo.

**Visualization:** Xuandong Jiang.

**Writing – original draft:** Shuang Guo.

**Writing – review & editing:** Xuandong Jiang.
